# Passive compliance or active innovation: The diffusion of public sport policies in China

**DOI:** 10.1371/journal.pone.0320651

**Published:** 2025-04-03

**Authors:** Zhiliang Li, Jian Liu, Hailin Lu, Jingjing Zhou

**Affiliations:** School of Physical Education, Shenzhen University, Shenzhen, Guangdong, China; Yeungnam University, KOREA, REPUBLIC OF

## Abstract

As an important tool for governing, regulating, and safeguarding national sports, sports policy not only centralizes and expresses the state’s vision for sports but also advances the development of the sports economy, and distributes the benefits of social sports. Accordingly, we collect policy samples from 31 provinces of China. Adopting the central–local relationship perspective, we use the grey correlation analysis method to explore the intrinsic relationship between the factors influencing the diffusion of public sports policies and the degree of policy text reproduction by provincial governments. We aim to encourage provincial governments to innovate policy texts based on local development needs, geographic position, and resource capabilities, This will enhance the diffusion of public sports policies and contribute to national and global sports governance. The diffusion of China’s public sports policies is influenced by six secondary indicators: economic level, population size, sports organizations, public sports demand, central government policy pressure, and learning effect. The policy environment, shaped primarily by central government policy pressure and the learning effect, has the greatest influence on the reproduction of provincial public sports policy texts.

## Introduction

As an important tool for governing, regulating, and safeguarding national sports, sports policy both centralizes and expresses a country’s vision for sports, promotes the development of the sports economy, and addresses the distribution of the social benefits of sports [1]. A policy undergoes the process of formulation, implementation, evaluation, monitoring, and termination [2]. However, the diffusion of public policy represents the process by which innovative central policies are disseminated to and adopted by provincial governments [3]. In the context of Chinese policy formulation and implementation, this diffusion is characterized by a vertical transfer from central to provincial governments. In response to central directives, local governments exhibit two distinct approaches to policy adoption: passive compliance or active innovation. From a policy adoption perspective, the hierarchical structure of governance in China means that higher-level government directives significantly influence the extent, volume, and pace of policy adoption at the local level. On a deeper level, examining policy text reproduction reveals that provincial governments may engage in passive compliance, merely replicating and transmitting central policies [4]. Alternatively, they may exhibit active innovation, refining and adapting central policies based on local development needs—a process known as “policy text reproduction” [5]. However, provincial governments often lack innovative approaches in their reproduced policies; this results in a “policy mimicry” phenomenon, in which the transmitted policies may not align with local realities [6]. This discrepancy can significantly influence both the effectiveness and governance of national policies [7].

This study is grounded in the central-local government relationship. It uses the grey correlation analysis method to investigate the primary factors influencing the diffusion of public sports policies in China and the extent of policy text reproduction by provincial governments (i.e., the total number of policies issued by provincial governments). It aims to encourage policy innovation by provincial governments based on local development needs, geographic advantages, and resource capacities; this will enhance the diffusion of public sports policies. This research contributes theoretical and practical insights to both national and global sports policy governance. It addresses the following questions:

1. What factors most significantly influence the reproduction of public sports policy texts by provincial governments?
2. Do these factors lead provincial governments to favor passive compliance or active innovation?
3. Why and how do these factors influence the decision-making process of provincial governments in the context of policy diffusion?

## Literature review

### Conceptualization of policy diffusion

As a process of knowledge production, the study of policy diffusion originated in the United States and is widely applied in political science and public administration as a key theory of policy change [8]. Policy diffusion is defined as the process by which an innovation is communicated among members of a social system, through certain channels over time [9]. Furthermore, it allows an innovation policy to spread to its users [10]. The diffusion of an innovation policy requires two actors: a policy disseminator, who passes on new ideas, policies, and programs to a policy recipient, and the policy recipient themselves [11]. The diffusion of public policy occurs when a government’s decisions are influenced by other governments [12]. Scholarly attention to the phenomenon of policy diffusion adoption over time has increased [13,14]. Numerous concepts with overlapping boundaries have emerged, such as policy convergence, transfer [15], emulation [16], learning [17], and lesson learning [18]. Furthermore, All these involve the mobility or diffusion of policy innovations from government to government and indirectly have the same characteristics. Policy diffusion differs from it by characterizing diffusion as a continuum, encompassing all points within the system [19].

### Research history of policy diffusion

Policy diffusion emerged as a field of research in the 1960s, with American scholars initially focusing on policy adoption, exploring why particular states adopt certain policies and not others [20]. Such research in political science also pioneered the use of event history and regression analyses to empirically model policy diffusion across states, which led to the concept of a new diffusion paradigm [20]. Based on Walker’s research, Gray [21] examined the diffusion of multiple policies. In the area of government-incentive behavior, Welch and Thompson [22] proposed that policies with federal incentives diffused faster than those without incentives based on motivation theory. In 1990, some scholars integrated national characteristics with regional diffusion models, proposing the concept of national policy diffusion model transformation. This perspective highlighted the interaction between national and regional traits in the policy diffusion process, offering a new lens for multidimensional studies on policy diffusion [23]. In the same year, numerous studies focused on government implementation of national policies at the grassroots level [24–26]. However, these studies emphasized macro-level factors, such as spatial, temporal, and hierarchical dimensions of policy diffusion, with limited attention to the micro-level actions of local governments in adapting and updating policy texts according to local development needs.

### Theoretical characterization of policy diffusion

As a process that facilitates policy exchange [27], the diffusion of policy innovations involves policy transfer. However, policy transfer emphasizes transfers between political systems [15] and is the application of public policies, administrative arrangements, and institutions located in one spatio-temporal context to other such contexts [28]. In terms of spatial diffusion, the diffusion mode describes how policies spread geographically and is characterized by extensibility and migration [29]. Regarding the development process of policy diffusion in terms of time, space, and organizational hierarchy, an S-shaped curve exists in the time dimension, a “proximity effect” in the spatial dimension, and a “leader–follower” effect in the hierarchy dimension. The spatial dimension is characterized by a “proximity effect” and a “leader–follower” cascading effect within regions [30].

However, which forces drive policy diffusion in the spatial-diffusion process? In terms of political forces, central government organizations, national organizations, and policy promoters drive the diffusion of public policies [31]. When variety of innovative policies are adopted and diffusion occurs, policy diffusion mechanisms emerge. In terms of policy diffusion dynamics, two main types of models—internal decision and external influence models [32]—are distinguished by the learning effect of early policy adopters, economic competition among neighboring cities, strong promotion by state governments, and imitation among large cities [33]. These effects are summarized by the terms “learning,” “competition,” “coercion,” and “imitation,” respectively [34]. Learning, competition, coercion, and constructivist theories provide the theoretical basis for policy diffusion [12].

### Influencing factors of policy diffusion

Domestic and international policy diffusion studies, have primarily examined external and internal factors. The external environmental factors influencing local governments’ policy adoption are attributable to the policy pressures exerted by higher-level and peer governments [35]. This study categorizes these influences into two modes of policy diffusion: top-down vertical diffusion [36] and horizontal diffusion among peer governments [37]. In China’s pressure-driven system, higher-level authorities typically establish policy targets and performance evaluation criteria, requiring local governments to comply with central directives, mandates, or regulations. This dynamic, referred to as central government policy pressure [38], compels local governments not only to conform but also to implement policies promptly in response to central authority. Additionally, local governments may actively adopt and reproduce policy texts to attract the attention of higher authorities and secure political or economic incentives, thereby promoting policy diffusion [39]. Alongside vertical policy diffusion, horizontal learning effects among local governments play a crucial role. This process involves an interactive mechanism among peer governments. Successful experiences in adopting and implementing sports policies are observed and emulated through meetings, forums, and research exchanges; this enables local governments to adjust and optimize their policies, thereby minimizing the cost and risk of policy innovation [40]. Consequently, both top-down legitimacy pressures and horizontal learning between peer governments significantly influence local governments’ policy adoption [41].

Regarding internal factors, local governments’ policy diffusion often depends on factors such as economic capacity, governance quality, urban structure, and population size [2,42]. This is because local government officials’ promotion prospects are often tied to economic performance [43]. Economic development not only dictates local governments’ fiscal capacity but also implies an ability to allocate resources such as funding effectively to ensure policy implementation [44]. This grants them greater flexibility in policy adoption and innovation. Population size directly reflects the complexity and scope of policy implementation at the local level. The success of sports facilities and public sports policies often depends on the locality’s population size and characteristics; when investing in sports infrastructure, local governments must consider these aspects to meet residents’ needs [45].

In policy diffusion, sports organizations act as direct agents of policy by organizing, promoting, and advocating, thereby aiding policy implementation at the grassroots level and providing valuable feedback for policy optimization [46]. Sports organizations can foster community identity through activities, enhancing trust and collaboration between residents and local governments [47]. They play an important role in connecting governments and communities, promoting social integration [48]. As living standards rise, residents’ demands for health and recreational activities increase, directly prompting local governments to prioritize investments and attention to sports policies. Public demand sports has a dual effect on policy diffusion: on the one hand, strong demand motivates local governments to adopt and promote related policies [49]; on the other hand, it provides a foundation for local governments to reproduce policy texts in ways that align with local needs [50].

Scholars have increasingly recognized that policy diffusion is not only a transmission process but also a dynamic one in which local governments innovate and reproduce policies based on specific needs. Particularly in sports policy, local governments face diverse regional economic, social, and cultural contexts; this promps them to refine or modify central policies in the adoption process to reflect the effects of local endowments [51]. Based on the literature, we identify identifies six secondary indicators: economic level, population size, sports organizations, public sports demand, central government policy pressure, and learning effect. The current scholarship provides a theoretical basis and framework for selecting factors that influence policy diffusion. However it does not systematically explain how these factors guide local governments’ decisions around passive compliance or active innovation in the context of sports policy text reproduction. This study aims to expand upon policy diffusion theory by addressing this gap, offering a more nuanced understanding of local governments’ behavior in the diffusion process.

### Policy adoption and policy reproduction

Policy adoption is commonly used to analyze local governments’ behavior in implementing policies issued by the central government within the formal institutional framework. Existing studies have primarily examined how a government’s policy choices are influenced by higher-level or peer governments from the perspective of policy diffusion [40]. After the central government releases a policy, various local governments not only adopt it but also engage in innovative policymaking, updating, and refining policy text content [52]. However, most studies have viewed policy adoption as a passive process and overlooked the proactive adjustments or autonomous innovations in policy content made by local governments in policy content during adoption. Additionally, comprehensive studies of the sports sector are lacking, especially in terms of a systematic theoretical framework to explain which factors most significantly influence provincial governments’ policy text reproduction behaviors. This study considers the reproduction of public sports policy texts as a critical innovation within policy diffusion theory. It addresses gaps in the study of sports policy diffusion and offers a deeper understanding of local governments’ decision-making behaviors.

## Methodology

### Grey correlation analysis method

The grey correlation analysis method is a tool for evaluating and describing the relationships between factors. It is especially useful in systems with limited or uncertain data, as it reveals the degree of association among variables [53]. It also identifies and ranks the influence of various factors on a target variable, indicating which factors are most closely related to the target [54]. This method focuses on the association between factors rather than absolute values, which allows to assess relative influence even with incomplete data. By contrast, regression analysis typically requires a complete dataset and assumes a normal distribution, emphasizing causal relationships but facing limitations in handling complex dynamic interactions [55]. Although qualitative research can provide understanding, it struggles with quantifying factor impacts across large samples, potentially leading to imprecision. The grey correlation analysis method calculates the similarity between reference and comparison sequences to quantify their association [56]. Therefore, it is the most suitable method for this study.

Step 1: Construct the Reference Sequence and Comparison Sequence. We set the reference sequence of the system as *X*_*0*_, with the formula as follows:


$$X_{0}=\left\{x_{0}\left(t\right)|t=1,2,\ldots ,n\right\}$$

This is a time series, where *t* represents a specific year, and each *x*_*0*_ (t) indicates the value of the reference variable at time *t*. The *m* sequences to be compared with the reference sequence are set as *X*_*i*_, with the formula as follows:


$$X_{i}=\left\{x_{i}\left(t\right)|t=1,2,\ldots ,n\right\}\;\left(i=1,2,\ldots ,m\right)$$

where *m* represents the number of comparison sequences (i.e., the number of influencing factors), and *x*_*i*_ (t) is the observed value of the *i* comparison sequence at time *t*.

Step 2: Calculate the Difference Sequence. Define the difference sequence as Δ_*i(t)*_, with the formula as follows:


$$\Delta_{i\left(t\right)}=\left|x_{0}\left(t\right)-x_{i}\left(t\right)\right|$$

Using the above formula, we obtain *m* difference sequences Δ_*i(t)*_, where *t* = 1,2,…,*n*.

Step 3: Determine the Maximum and Minimum Difference Values. To ensure comparability on a unified scale in the following steps, we calculate the maximum and minimum values of the difference sequences as follows:


$$\Delta_{max}=max_{i,t}\;\Delta_{i\left(t\right)}\Delta_{min}=min_{i,t}\;\Delta_{i\left(t\right)}$$

The formula identifies the maximum and minimum difference values across all sequences *i* and time points *t*.

Step 4: Calculate the grey correlation coefficient, which indicates the similarity between the reference sequence *X*_*0*_ and each comparison sequence *X*_*i*_ at each time point, using the following formula:


$$\xi_{i}\left(t\right)=\frac{\Delta min+\rho \Delta max}{\Delta_{i\left(t\right)}+\rho \Delta max}$$

The parameter *ρ* is the distinguishing coefficient, which typically takes values in the range [0,1] [57].

In recent years, numerous scholars have applied the grey correlation analysis method to fields such as sports tourism, the sports industry, and the digital economy [44,58,59], demonstrating its utility. However, in the study of sports policy diffusion, this method has not yet been widely adopted. Current research in sports policy tends to rely on qualitative analysis or traditional quantitative methods, which often struggle to capture the complex interactions between factors in policy diffusion processes. This study employs the grey correlation analysis method [57] to explore the correlation between provincial policy text reproduction and the factors influencing public sports policy diffusion. It offers a new perspective for quantitatively assessing complex dynamic relationships.

### Design of data indicators

Policy diffusion research has focused on the process whereby one entity adopts an innovative policy introduced by another. Consequently, the factors influencing the diffusion of China’s public sports policies should reflect the elements that affect local governments’ policy adoption. This study employs a two-tier indicator system to represent the policy diffusion process on various levels (see Table 1).

**Table 1:** Factors influencing the proliferation of the main public policies in the field of sports.

Tier 1 Indicators	Secondary indicators	Indicator data sources
Policy subjects	Economic level	Annual GDP((CNY) 100 million)
	Population size	Total population at the end of the year (10,000 persons)
Policy objects	Sports organizations	Number of sports organizations per year
	Public sports demand	Share of urban population to total population at the end of the year
Policy environment	Central government policy pressure	(Number of central policies adopted by provincial governments / Total number of central policies) × (Total number of provincial policies / Total number of central policies)^(-α)
	Learning effect	The ratio of the number of provinces that have adopted central public sports policy texts to the total number of provinces

We derive the primary indicators in this research—policy subjects, policy objects, and policy environment—are derived from established policy science frameworks. Policy subjects are individuals, groups, or organizations directly or indirectly involved in policy formulation, execution, evaluation, and monitoring [5]. As the subjects of provincial governments directly shape policy reproduction, this research identifies provincial governments as the primary policy actors [60]. Policy objects are the entities affected by policies and play a key role in policy acceptance, implementation, and feedback [61]. Within policy diffusion, policy objects need to be covered by the policy and should actively engage and respond positively to its implementation. The policy environment encompasses all factors that influence the policy’s development, existence, and progression [62]. In China’s pressure-driven system, both higher-level and peer governments influence provincial governments’ policy behaviors, facilitating or hindering broader policy diffusion.

We develop the secondary indicators of this study are developed based on the primary indicators, which include economic level, population size, sports organizations, public sports demand, central government policy pressure, and learning effect.

The study designates economic level (annual GDP) and population size (end-of-year population total) as secondary indicators under the primary indicator of “policy actor”. GDP, representing overall economic strength, provides a comprehensive reflection of the local government’s economic status and policy implementation capacity, more so than other economic indicators such as per capita income or fiscal expenditure, it thus offers a macro-level perspective [63]. Population size reflects the scope of public service demand and the extent of resource allocation pressures on local governments. Using year-end population totals enables tracking dynamic changes over time, thus showing the pressures faced by local governments in terms of resource distribution, infrastructure, and policy execution [64].

Sports organizations (annual number of sports organizations) and public sports demand (ratio of urban population to total year-end population) are included as secondary indicators under the policy targets. The number of sports organizations is an important indicator of community engagement [65]. A high number signifies a robust network linking local governments and grassroots society. Urban population ratio reflects changes in geographic population distribution and indicates potential demand intensity for sports facilities and activities, with higher urbanization indicating stronger public demand for sports services. Population size provides a basis for policy coverage; however, public sports demand is a driving force influencing policy adaptation and reproduction, representing both the breadth and depth of policy diffusion.

“Central government policy pressure” represents the influence exerted by the central government on provincial governments to ensure compliance with higher-level requirements and objectives. It is measured by the volume of policies issued by the central government and the 31 provincial governments. Simply calculating the adoption ratio may lead to imbalance as the central government releases fewer policies each year, whereas provincial governments adopt and issue them in large quantities. Therefore, we introduce an adjustment factor is introduced to account for the frequency of policy issuance, balancing central policy output and provincial policy adoption, resulting in a more accurate measure of the central government’s policy pressure. The calculation formula for central government policy pressure is as follows:


$$Centralgovernmentpolicypressure=\frac{\begin{array}{c} NumberofPoliciesAdopted\\ byProvincialGovernments \end{array}}{NumberofCentralPolicies}\times {\left(\frac{\begin{array}{c} TotalNumberofPolicies\\ byProvincialGovernments \end{array}}{NumberofCentralPolicies}\right)}^{-\alpha }$$

The central government issued 10 policies, which were adopted 172 times by provincial governments, while the total number of policies issued by provincial governments was 249. The parameter α is set to 0.5 by default, striking a balance by neither over-sensitizing the model to differences nor excessively smoothing it. This setting allows for an objectively reflecting on the relationships between different policies. Using −α reduces the influence of high provincial policy counts on central government policy pressure, ensuring that the significance of the latter is not diminished owing to an increase in the denominator.

Cross-regional policy diffusion research has recognized the learning effect as a major driving force. By analyzing and observing policy effects in neighboring or similarly developed areas, regions can better align their policies with local needs and circumstances. The formula for calculating the learning effect is as follows:


$$LearningEffect=\frac{\begin{array}{c} NumberofProvincesAdopting\\ CentralSportsPublicPolicies \end{array}}{TotalNumberofProvinces}$$

### Data sources and calculation procedures

This study uses the total number of reproduced public sports policy texts in China’s 31 provinces from 2016–2021 as a benchmark, including both policies adopted from central government initiatives and independently developed innovations. Covering all provinces, autonomous regions, municipalities, and special administrative regions, this scope ensures diverse sample representation across economic levels, enhancing the study’s relevance. The period from 2016 to 2021 marks a pivotal transition in Chinese sports policy, from focusing on public health to enhancing international athletic competitiveness, aligning with the dual goals set in the Healthy China 2030 Planning Outline. Following the 2021 Tokyo Olympics, this timeframe reflects not only the impact of these strategies but also the intensified domestic policy innovation; it thus provides a valuable window for observing the sustained effects of these sports policies.

In the grey correlation analysis method, the primary sequence (reference sequence) represents the main metric in the study, typically reflecting an overarching phenomenon to provide a baseline for comparison. The auxiliary sequences (comparative sequences) are related indicators that influence the primary sequence. This study designates the extent of reproduced public sports policies in 31 provinces from 2016 to 2021 (total policies issued by provincial governments) as the reference sequence. It sets each year’s secondary indicators, which capture factors influencing the diffusion of public sports policies, as comparative sequences.

The data for the reference sequence in this study are sourced from the 2024 Sports Policy Directory published by the General Administration of Sport of China, accessible at https://www.sport.gov.cn/gdnps/files/c28052072/28156549.pdf, as well as from the official portals of the 31 provincial governments (linked through the national government website: https://www.gov.cn) and of provincial sports departments (accessible at https://www.sport.gov.cn).

For the comparative sequences, data on economic level, population size, and public sports demand are derived from the China Statistical Yearbook (https://www.stats.gov.cn/sj/ndsj/), while information on sports organizations is available from the General Administration of Sport of China (https://www.sport.gov.cn). Data for central government policy pressure and learning effect are sourced from both the national government portal (https://www.gov.cn) and the provincial government and sports department websites.

The analysis steps are as follows: First, we establish the reference and comparative sequences functions are established. Second, we perform data normalization by calculating the mean of each data group and dividing each original value by this mean; therefore, we achieve a uniform scale across indicators and eliminating unit discrepancies. Third, we calculate the absolute differences between the reference and comparative sequences at each time point are calculated. Fourth, we process these differences through the grey correlation coefficient formula to derive correlation values. Finally, we determine the overall correlation degrees are determined from these values, This allows us to calculate each influencing factor’s final correlation degree [66].

## Analysis of results

Step 1 involves setting the total number of public sports policy texts reproduced annually from 2016 to 2021 by 31 provinces as the reference sequence, The secondary indicators of the influencing factors on public sports policy diffusion in China from 2016 to 2021 serve as the comparative sequences (Table 2).

**Table 2:** Parent–child series of secondary indicators of impact factors and degree of reproduction of policy texts.

Year	Total	Economic level	Population size	Sports organizations	Public sports demand	Central government policy pressure	Learning effect
2016	63	746395.1	139232	47280	58.84	0.567	0.290322581
2017	69	832035.9	140011	48000	61.50	6.501	0.935483871
2018	15	919281.1	140541	53750	61.50	2.324	0.290322581
2019	19	986515.2	141008	49092	62.71	2.92	0.516129032
2020	36	1013567	141212	47300	63.89	4.007	0.709677419
2021	47	1141230.8	141260	60176	64.72	7.002	0.935483871

Step 2 involves normalizing the data using IBM SPSS Statistics Version 27. First, we import the raw data from 2016–2021 were imported into SPSS, ensuring that all values exceeded zero. Next, under the “Analyze” menu, we select “Descriptive Statistics” and chose “Descriptives” to calculate the mean for each variable. Finally, we employ the “Compute Variable” function was employed to generate normalized variables, standardizing values around 1. This approach facilitates comparison across variables of differing magnitudes (Table 3).

**Table 3:** Meanized series of secondary indicators of impact factors and degree of reproduction of policy texts.

Year	Total	Economic level	Population size	Sports organizations	Public sports demand	Central government policy pressure	Learning effect
2016	0.72	0.53	0.59	0.57	1.37	1.04	0.72
2017	0.78	0.67	0.72	0.67	0.01	0.14	0.78
2018	0.62	0.64	0.69	0.63	0.24	0.11	0.62
2019	0.59	0.55	0.51	0.55	0.29	0.38	0.59
2020	0.21	0.14	0.06	0.16	0.16	0.29	0.21
2021	0.08	0.13	0.05	0.09	0.67	0.39	0.08

The normalization formula applied is as follows:


$$NormalizedVariable=\frac{OriginalVariable}{Mean}$$

Step 3 involves calculating the absolute difference between each comparative sequences and the reference sequence at the same time point, with the corresponding difference values displayed in Table 4. A smaller absolute difference indicates a higher degree of similarity, which reflects a stronger correlation. This step avoids the complexity associated with handling negative differences; it aims to quantify the similarity between the comparative and reference sequences, thereby assessing their correlation.

**Table 4:** Series of absolute differences between the reference sequence and comparative sequences at the same moment in time.

Year	Economic level	Population size	Sports organizations	Public sports demand	Central government policy pressure	Learning effect
2016	0.72	0.53	0.59	0.57	1.37	1.04
2017	0.78	0.67	0.72	0.67	0.01	0.14
2018	0.62	0.64	0.69	0.63	0.24	0.11
2019	0.59	0.55	0.51	0.55	0.29	0.38
2020	0.21	0.14	0.06	0.16	0.16	0.29
2021	0.08	0.13	0.05	0.09	0.67	0.39

Step 4 involves substituting the corresponding difference values, along with the minimum and maximum differences, into the formula to calculate the grey correlation coefficient (see Table 5). In this case, Δmin =  0.01, Δmax =  1.37, and *ρ* =  0.1. The grey correlation analysis formula used is as follows [67]:

**Table 5:** Grey correlation coefficients between secondary indicators of impact factors and degree of reproduction of policy texts.

Year	Economic level	Population size	Sports organizations	Public sports demand	Central government policy pressure	Learning effect
2016	0.17	0.22	0.20	0.21	0.10	0.12
2017	0.16	0.18	0.17	0.18	1.00	0.54
2018	0.20	0.19	0.18	0.19	0.39	0.59
2019	0.20	0.22	0.23	0.21	0.34	0.28
2020	0.42	0.54	0.74	0.50	0.49	0.34
2021	0.67	0.56	0.79	0.64	0.18	0.28


$$Li\left(t\right)=\frac{\Delta min+\rho \Delta max}{\Delta_{i\left(t\right)}+\rho \Delta max}$$

This step enables the determining of the influence strength and closeness of each factor to the target variable, helping identify the most impactful factors. Δi(t) represents the absolute difference between each comparative sequences and the reference sequence at the same time point. For instance, in Table 4, the Δi(t) for Economic Level is 0.72, which, when applied to the grey correlation coefficient formula, yields a corresponding coefficient of 0.17 in Table 5.

The minimum absolute difference, Δmin, between the comparative and reference sequences across all time points, is included in the numerator to ensure that the similarity measure remains positive, thereby preventing extreme values. The maximum absolute difference, Δmax, standardizes the scale of differences, constraining the variations of all comparative sequences within a relatively uniform range. This allows the model to handle varying magnitudes consistently and enhances the stability of the correlation calculations.

In this study, we select a resolution coefficient of ρ =  0.1 to modulate the influence of Δi(t) on the correlation degree, thus ensuring a smoother calculation across different time points. First, ρ =  0.1 enhances the sensitivity to subtle differences, improving interpretability and applicability. Second, it aligns with the sensitivity requirements of grey system theory, revealing hidden correlations within complex systems [68]. Finally, empirical validation indicates that ρ =  0.1 produces results that align well with expected patterns, enhancing precision and practical value.

In the formula’s denominator, Δi(t)+ρΔmax functions as a normalization measure, preventing large fluctuations in the correlation calculation due to extreme values. The resolution coefficient ρ is introduced to finetune the model’s sensitivity to minor variations; a smaller ρ makes the model more responsive to differences. The calculated *Li(t)* value represents the correlation degree between the comparative and reference sequences at time *t*, where values closer to 1 indicate stronger similarity and correlation, and lower values indicate weaker association.

Table 4 show that the absolute differences for central government policy pressure in 2016 and 2017 are 1.37 and 0.01, respectively, representing the largest and smallest values among all factors. This indicates a weaker correlation in 2016 and a stronger correlation in 2017. Analyzing these absolute differences show that both central government policy pressure and learning effects exhibit considerable deviations from the target variable; this suggests that they have complex impacts on policy diffusion. Overall, Table 4 provides essential foundational data for calculating the grey correlation coefficients in Table 5.

Step 5 involves calculating the grey relational grade. We sum and average the correlation coefficients for each secondary indicator of the factors influencing sports policy diffusion from 2016 to 2021. This provides the grey relational grade between each secondary indicator and the total number of provincial policy reproductions. Subsequently, We aggregate the grey relational grades of secondary indicators to determine the relational grades of policy subject, policy object, and policy environment in relation to the reproduction extent of public sports policies (Table 6). This step consolidates and assesses the relational grades calculated in the previous step, providing a comprehensive overview of how these factors collectively influence the target variable.

**Table 6:** Grey correlation between the indices of primary and secondary indicators of impact factors and the degree of reproduction of policy texts.

Policy subjects		Policy objects		Policy environment	
Economic level	Population size	Sports organizationss	Public sports demand	Central government policy pressure	Learning effect
0.30	0.32	0.39	0.32	0.42	0.36
0.62		0.71		0.78	

The grey relational coefficients in Table 5 represent the strength of association between each secondary indicator and the extent of provincial government policy text reproduction. These coefficients range from 0 to 1; where values closer to 1 indicate a stronger correlation between the influencing factor and policy text reproduction, whereas lower values suggest a weaker association.

The results in Table 5 show that the grey relational coefficient for central government policy pressure in 2017 is 1.00. This indicates a particularly very strong association between central government policy pressure and provincial policy text reproduction that year, with central policies significantly driving provincial government actions. In contrast, the learning effect in 2016 has a grey relational coefficient of 0.12; this suggests a lower influence, reflecting minimal reliance on past policy experiences by provincial governments. Over multiple years, factors such as economic level, sports organizations, and public sports demand exhibit relatively high and increasing grey relational coefficients. This indicates their substantial and growing impact on provincial policy reproduction. This trend underscores the continuous regional efforts to enhance economic development, manage sports organizations, and meet public sports needs.

In the grey correlation analysis method, correlation coefficients typically lack the traditional significance test p-values [54]. However, the magnitude of these coefficients can serve as an indicator of significance. As shown in Table 6, this study sets 0.4 as the threshold for strong correlation, with coefficients above 0.4 considered to have a significantly influence the extent of provincial governments’ policy text reproduction.

From a micro perspective, the order of correlation strengths between the secondary indicators of factors influencing sports policy diffusion in China and provincial governments’ reproduction of public sports policy texts is as follows: Central government policy pressure (0.42)>  sports organizations (0.39)>  learning effect (0.36)>  public sports demand (0.32), population size (0.32)>  economic level (0.30). Central government policy pressure, with a correlation coefficient of 0.42, is the only factor surpassing the significance threshold of 0.4. Sports organizations follow closely with a correlation of 0.39. Meanwhile, the economic level, with the lowest correlation coefficient of 0.30, indicates that although economic level and population size are essential contextual factors for local policy implementation, they are not as directly influential as central government policy pressure and sports organizations in driving policy reproduction.

From a macro perspective, the order of correlation strengths between the primary indicators of factors influencing sports policy diffusion in China and the provincial governments’ public sports policy texts is as follows: Policy environment (0.78)>  policy object (0.71)>  policy subject (0.62). First, the policy subject, which includes economic level and population size, is the least influential factor. Second, the policy object plays a secondary role in the reproduction process. While provincial governments consider local social structures and public needs, this factor’s influence remains weaker than that of central policies’ directive power. Finally, the external policy environment correlates the strongest with policy text reproduction. Under the dual influence of central government policy pressure and learning effects, policy diffusion exhibits characteristics of both vertical concentration and horizontal circulation.

## Discussion

With the widespread adoption of global sports governance principles, nations are actively developing and implementing public sports policies to facilitate their diffusion and achieve specific policy objectives. The following analysis will examines the specific impacts of primary and secondary indicators on the diffusion and reproduction of public sports policies, exploring the underlying decision-making mechanisms at work.

First, the findings indicate that the secondary indicator of central government policy pressure (correlation coefficient 0.42) plays a dominant role in the diffusion of policies from central to local governments. Specifically, in the context of China’s New Whole-Nation System, the central government exerts policy pressure on provincial governments by issuing policy guidelines, establishing implementation requirements, and setting specific performance indicators. This top-down pressure mandates provincial governments to implement policies swiftly—particularly those that are crucial to economic development and national strategic interests. Owing to the inherently strong goal-orientation of central government policies, provincial governments often adopt a passive compliance strategy in the policy adoption process. This means prioritizing the adoption of policies that are highly emphasized by the central government, ensuring alignment with the central policy direction. This aligns with the top-down characteristics of policy diffusion theory [69], where legitimacy pressure from higher levels prioritizes compliance, limiting provincial innovation. Additionally, sports organizations (0.39) and learning effects (0.36) significantly influence policy text reproduction. Sports organizations provide localized information, distribution channels, and resources, enabling provincial governments to better understand and respond to regional needs. Meanwhile, provincial governments also engage in horizontal learning with their counterparts, promoting the flexible adaptation of policies. This aligns with the “learning mechanism” in policy diffusion theory [33], whereby provincial governments improve policy diffusion through mutual learning and exchange.

Second, the results of this study show that the impact of economic development on the provincial government’s policy text reproduction is relatively weak, which contrasts with some studies suggesting that higher economic levels promote the diffusion of policies [42]. While we agree that higher economic levels can facilitate policy adoption [70], provincial governments’ policy decisions are shaped by a wider range of micro-level factors, such as top-down assessment incentives and competition for resources among peer governments [71]. Moreover, the adoption of sports policies requires a broader political, social, and institutional context. Even in economically developed regions, sports policies often receive lower priority compared to more pressing issues like healthcare and education. Therefore, economic development does not necessarily lead to the adoption of sports policies.

Third, among the primary factors, provincial governments are most influenced by the policy environment. Decision-makers at the provincial level must prioritize information to address the structural conflict between information overload and attention limitations. This “disproportionate information processing” leads to an imbalanced attention allocation [72]. Therefore, provincial decision-makers focus on central mandates and performance targets, aiming to maintain accountability within the bureaucratic hierarchy [73]. Noncompliance with central mandates often results in accountability pressures [74], diminishing the impact of other factors. However, when a province innovates and achieves favorable policy outcomes, it triggers a learning mechanism that significantly influences policy reproduction in other provinces. For instance, after Jiangsu’s “River Chief System” pilot policy gained central attention [52], a bottom-up diffusion mechanism emerged, prompting national policy adoption [75].

Finally, within China’s sports governance framework, this study supports a flexible centralization model. Provincial governments use sports organizations as intermediaries for policy implementation, incorporating feedback mechanisms that allow them to adapt central policies into local innovations after initial compliance. This flexible model transcends the traditional “top-down” policy diffusion approach, granting provincial governments more autonomy to dynamically dynamically tailor policies to local conditions. It thus provides a new perspective on China’s governance model.

In the context of global sports governance, this study promotes an adaptive innovation mechanism. Despite limited direct influence from economic factors, local governments can bridge resource gaps through collaborations with social organizations and intergovernmental learning. This approach is especially relevant for resource-constrained developing countries, where sports policies can be customized and innovatively diffused by leveraging local partnerships and cross-governmental insights. Therefore, global governance would benefit from empowering local governments with the flexibility to foster localized innovation and adapt policies for sustained and effective implementation.

## Conclusion

This study systematically analyzed from the central–local government relationship perspective, how various factors influence the extent of provincial governments’ policy text reproduction during the diffusion of public sports policy in China. It aimed to deepen provincial governments’ understanding of policy innovation and encourage its adaptation. Moreover, different demands from provincial governments may sometimes conflict [76], and notable distinctions exist between top-down and horizontal diffusion [77]. Top-down diffusion involves central government interventions that motivate or constrain policy adoption at the provincial level, which result in incentive or constraint effects [78]. In contrast, horizontal intergovernmental diffusion typically reflects learning or competitive dynamics [79]. However, this study focused solely on the learning perspective when examining horizontal intergovernmental relationships.

This study examined the factors that most significantly influence provincial governments’ reproduction of public sports policy texts. It analyzed three primary indicators—policy subject, policy object, and policy environment—and six secondary indicators—economic level, population size, sports social organizations, public demand for sports, central government policy pressure, and learning effect. Considering the nature of policy diffusion in China’s unitary system, this study further explored the top-down mechanisms as the dominant drivers of policy reproduction at the provincial level as well as bottom-up mechanisms that facilitate broader dissemination.

This study explored why and how these primary and secondary indicators influence the reproduction of public sports policy texts by provincial governments. Within China’s pressure-driven system, the process follows a “focus–motivation–execution” logic, in which factors of varying degrees of correlation affect the allocation of intergovernmental attention. This, in turn, plays a critical role in setting policy agendas, driving the diffusion of policies.

This study, using grey correlation analysis, found that although provincial policy text reproduction is constrained by pressure from the central government, provincial governments can enhance political performance by strengthening policy innovation. However, the current policy environment leans toward passive compliance, which may inhibit local governments’ proactive efforts in policy customization and innovation. Future national policy design and implementation should focus on how to encourage and support local governments in exploring locally tailored solutions, ensuring both policy consistency and adaptability for national effectiveness.

## Limitations and future research

First, current policy diffusion research focuses on whether specific policies are adopted and on the speed of adoption [80]; this aligns with the assumption of this study that the intensity of policy implementation is consistent once policies are adopted by local governments. However, owing to varying levels of influence from different factors on provincial governments’ reproduction of sports policy texts, this study did not account for regional differences across Chinese provinces. Future research could incorporate regional disparities to further analyze individual differences in provincial policy diffusion and reproduction, gaining a better understanding of local governments’ behavior under varying conditions.

Second, this study primarily examined factors such as economic level, sports organizations, and learning effects in the context of policy text reproduction. However, in practice, additional potential factors, such as the decision-making preferences of local officials, cultural differences, and public participation, may also play substantial roles. Future research should expand the range of variables and consider perspectives from policymakers and policy recipients, allowing for a more comprehensive understanding of the complex process of policy text reproduction.

## Supporting information

S1 File Data appendix. (XLSX)
